# Supplementary figures and images for: Current pattern of antibiotic resistance and molecular characterization of virulence genes in Klebsiella pneumoniae obtained from urinary tract infection (UTIs) patients, Peshawar
Source: PLoS One. 2025 Apr 10;20(4):e0319273. doi: 10.1371/journal.pone.0319273 (PMC11984708; doi:10.1371/journal.pone.0319273)

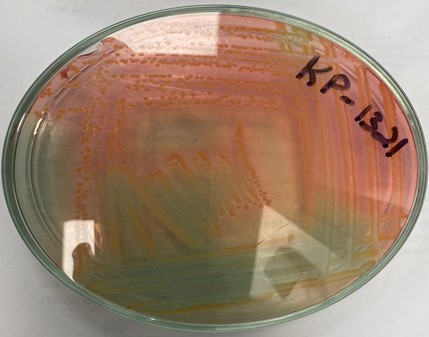

Supplement: S1 Fig — (JPG) [file pone.0319273.s001.jpg]

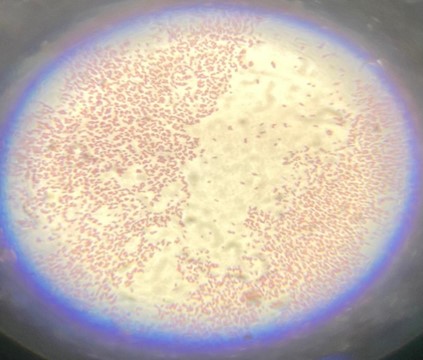

Supplement: S2 Fig — (JPG) [file pone.0319273.s002.jpg]

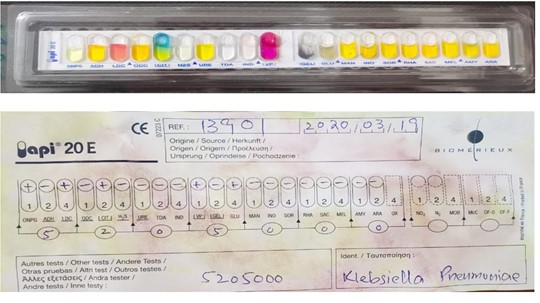

Supplement: S3 Fig — (JPG) [file pone.0319273.s003.jpg]

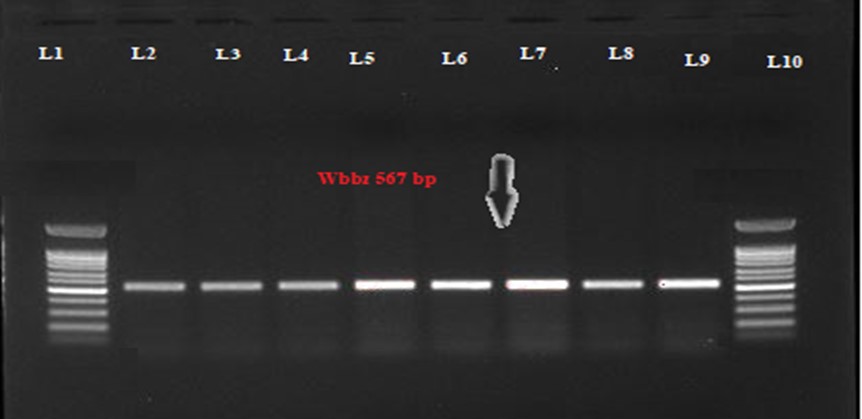

Supplement: S4 Fig — (JPG) [file pone.0319273.s004.jpg]

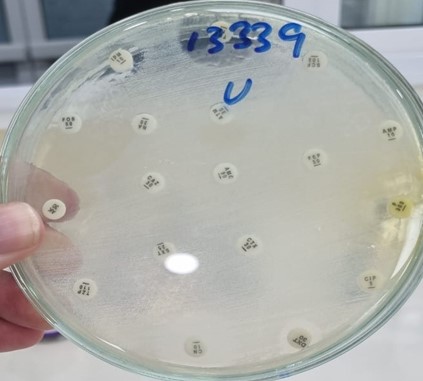

Supplement: S5 Fig — (JPG) [file pone.0319273.s005.jpg]

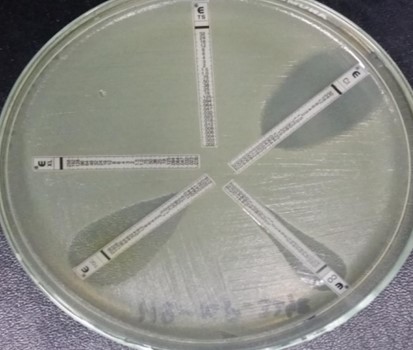

Supplement: S6 Fig — (JPG) [file pone.0319273.s006.jpg]

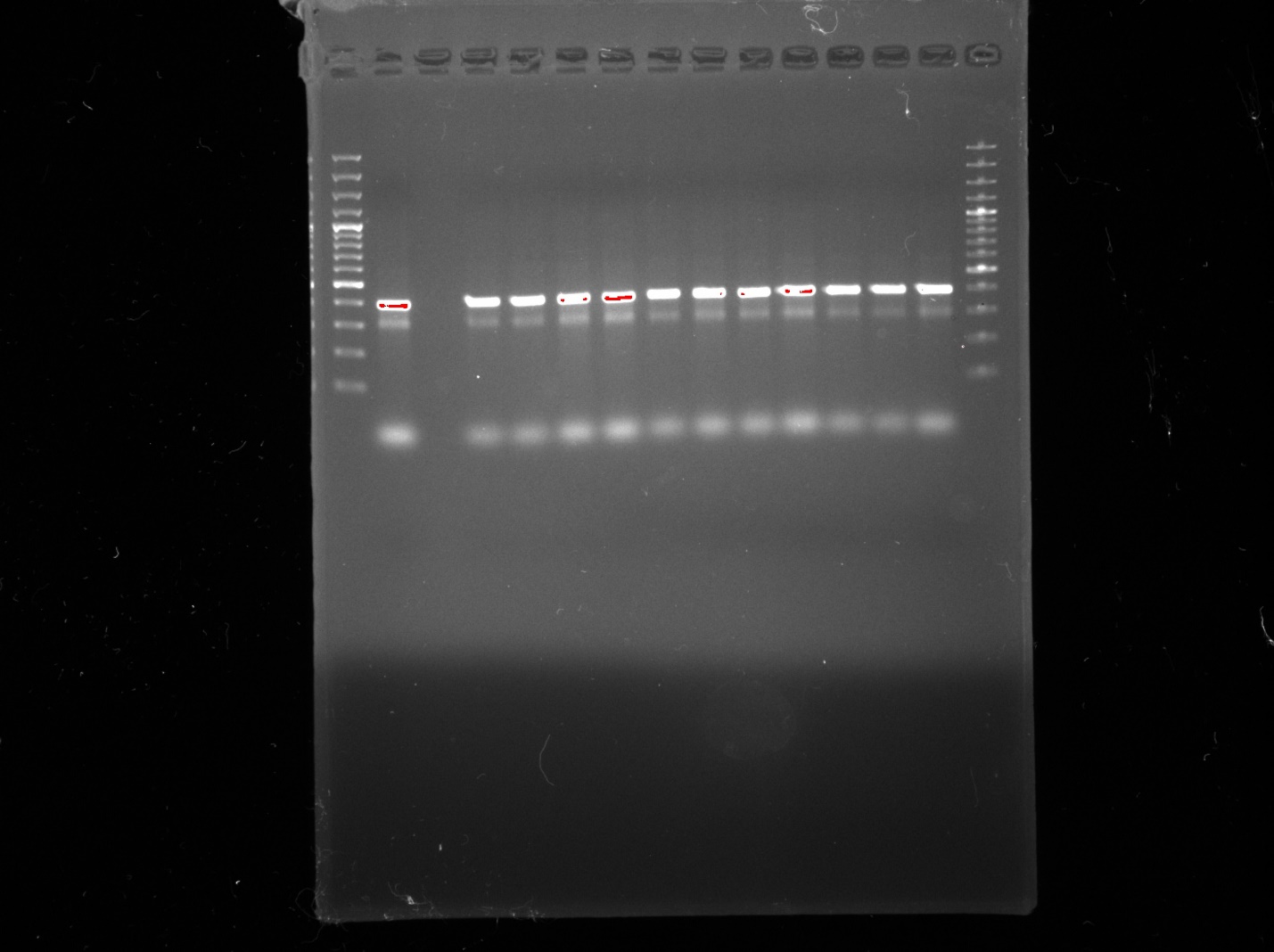


**A**


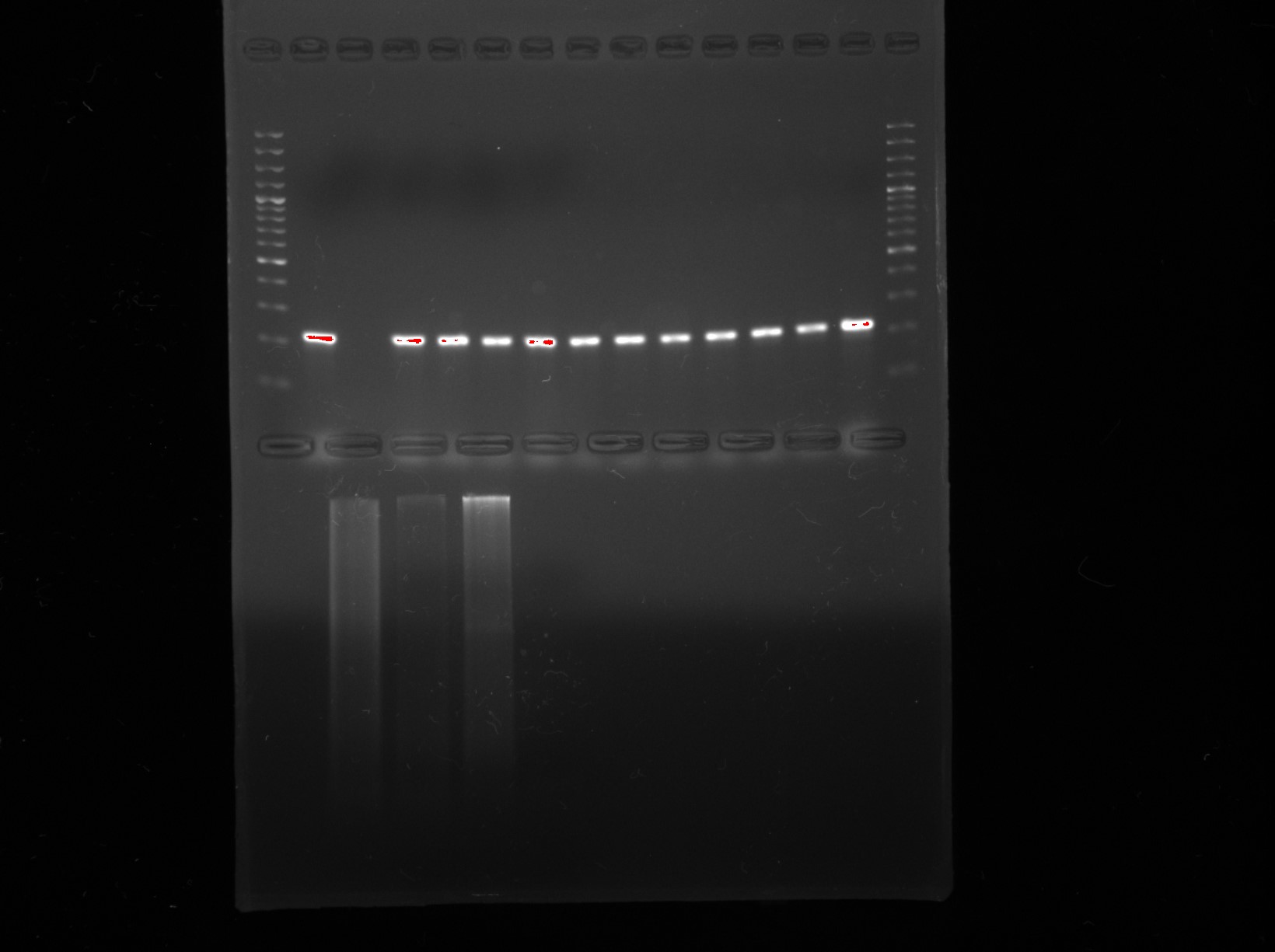


**B**


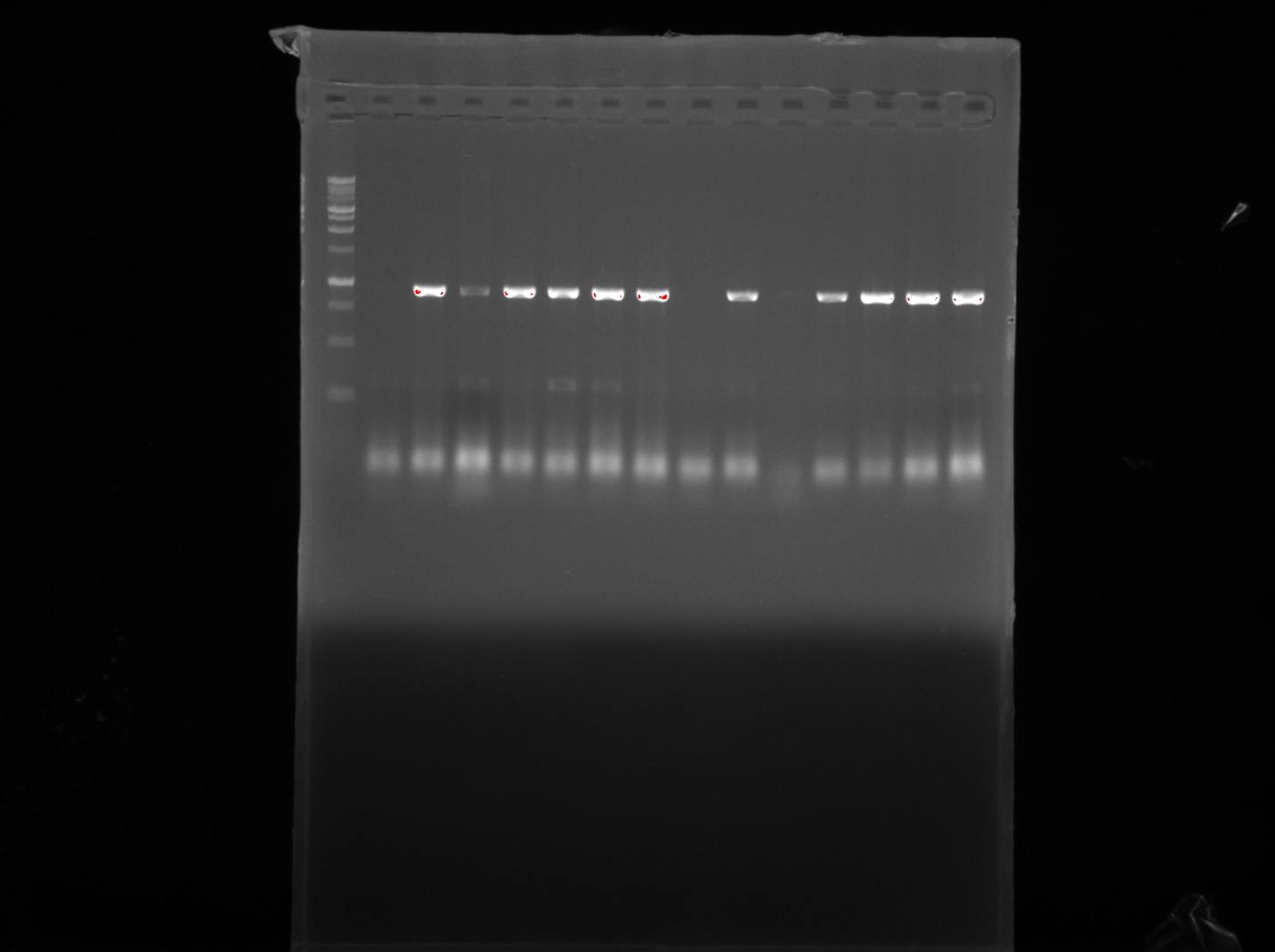


**C**


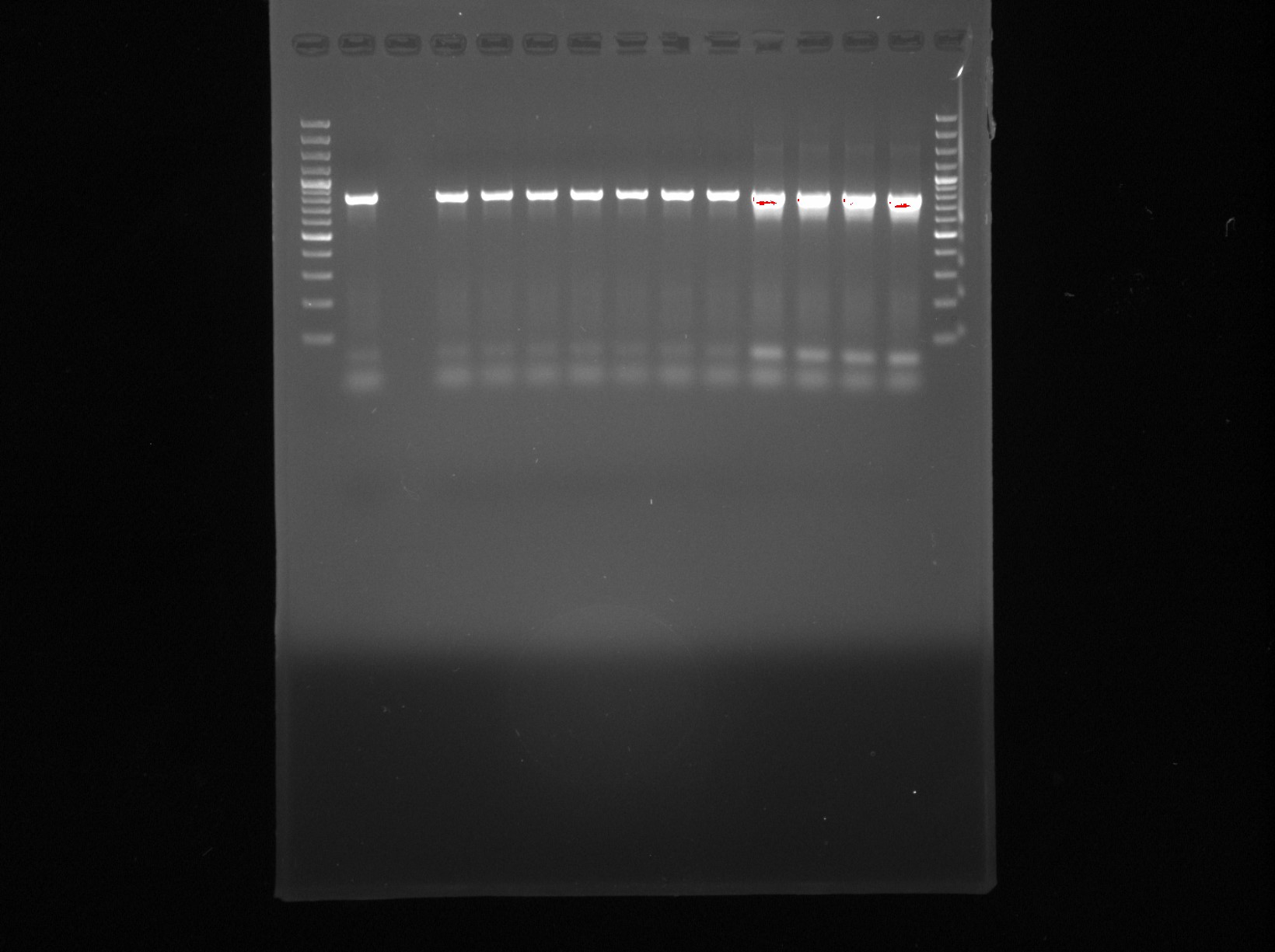


**D**


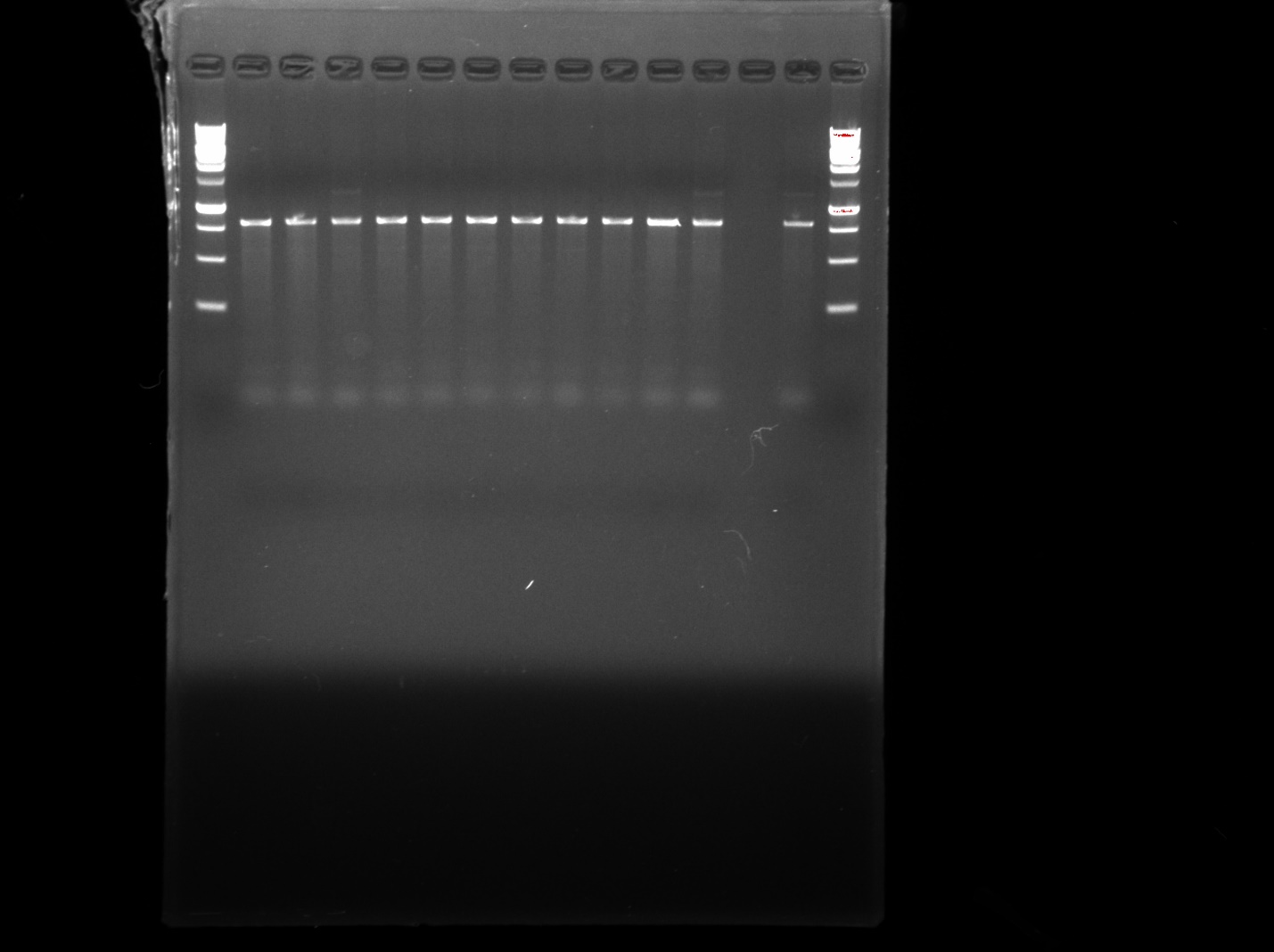


**E**

**S7 Fig:** Original uncropped images of gel of figure 2 of the main text

Supplement: S7 Fig — (DOCX) [file pone.0319273.s007.docx]
